# Isotope Effect on the Hydrogen Ordering from Ice V to Ice XIII via a Partially Ordered Intermediate

**DOI:** 10.1021/acs.jpcb.6c01462

**Published:** 2026-06-13

**Authors:** Keishiro Yamashita, Thomas Loerting

**Affiliations:** † Institute of Physical Chemistry, 27255 University of Innsbruck, Innrain 52c, 6020 Innsbruck, Austria; ‡ SUPA, School of Physics and Astronomy and Centre for Science at Extreme Conditions, 3124 The University of Edinburgh, Edinburgh EH9 3JZ, U.K.

## Abstract

Although ice polymorphs commonly feature orientational order and disorder, it is difficult to grasp the nature of partial order. In this study, we report on the hydrogen ordering of ice V using calorimetry at ambient pressure with an isothermal annealing approach. H_2_O/D_2_O isotopic substitution underlines the existence of the partially ordered intermediate state between ice XIII (below 113 K) and ice V (above 120 K), which exhibits a large isotope effect on the enthalpy of hydrogen disordering. Combined with the observation of two-staged time evolution of hydrogen order and the significant deuteration-induced slowdown of the ordering kinetics by a factor of 15–60, we propose that this intermediate state bears dynamic disorder. This reflects mutual conversions of ordered configurations taking place, i.e., domain fluctuations between differently ordered configurations. This finding raises a new perspective to characterize partial order, leading to the potential application toward frustrated functional materials.

## Graphical abstract

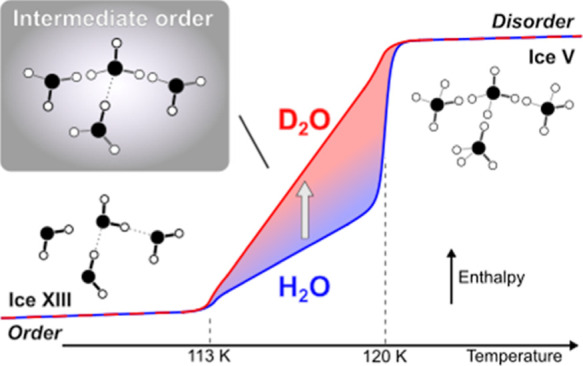

## Introduction

The order–disorder transition is a ubiquitous phenomenon observed in various crystalline materials, famously in water ice. The disordered structure is also termed the geometrically frustrated structure, which is associated with residual entropy even at very low temperatures, known as configurational entropy.^1^ Such a geometrical frustration is sometimes not released spontaneously, but this has been achieved for at least six different polymorphs with various approaches.^2–4^ The structural modification upon ordering and releasing the geometric frustration often entails drastic changes in physicochemical properties such as (anti)­ferroelectricity,^5–7^ magnetism,^8^ rheology,^9^ chemical transportation,^10^ and local dynamics.^11–13^ These have impacts on fields of geo- and planetary science and biology, as well as industrial applications. Nevertheless, our understanding is far from complete, even or especially for water ice, one of the simplest molecular systems.

Water ice polymorphs feature order–disorder transitions on the hydrogen sublattice, i.e., the molecular dipoles reorient at the transition. The ordered low-temperature phase shows low relative permittivity ε_r_ (typically ≈2–3), while the disordered counterparts are high-ε_r_ phases, typically with ε_r_ > 100.^14–16^ On this transition, the oxygen sublattice is retained, which represents the framework for the hydrogen-bonded network topology. Changes in the oxygen sublattice are accompanied by drastic network changes and are associated with enthalpy differences up to ≈2700 J mol^–1^.^17^ By contrast, energy differences among various H-sublattice configurations are usually small, within the range between 10 and 300 J mol^–1^,^18,19^ so that they tend to mix up at finite temperatures.^7,20^

Owing to the small enthalpy difference, water molecules can orient randomly at temperatures above a transition temperature (*T*_tr_) where the contribution of the configurational entropy (*S*_conf_) compensates for the enthalpic disadvantage. Thus, the hydrogen-disordered state can be represented by a mixture of different orientational arrangements, i.e., molecular configurations. On the other hand, a handful of ordered low-enthalpy structures might appear below *T*_tr_. The value of *S*_conf_ is zero for the single completely ordered form with the lowest enthalpy, in contrast to nonzero *S*_conf_ for disordered phases. A simple approximation provided by Linus Pauling^1^ estimates *S*_conf_ = *R* ln­(3/2) = 3.37 J mol^–1^ K^–1^ with the gas constant (*R*) for the completely disordered structure. This estimate relies on the constraints provided by the Two-in/Two-out Bernal-Fowler ice rules.^21^ Despite its simplicity, the deviation of *S*_conf_ from more realistic models taking into account the geometrical constraints from hydrogen-bond topology is only 1–2%,^22–25^ so that the Pauling entropy is used even today to evaluate the degree of order.

Since the discovery of the first hydrogen order–disorder transition for the polymorph pair ice VII–VIII in 1966,^26^ this simple phenomenon has been studied for over half a century for ordinary ice I_h_ ^18^ and six different high-pressure ice polymorphs.^3,27–29^ Typical analytic techniques include diffraction,^4^ calorimetry,^19,30^ dielectric spectroscopy,^12,13^ and vibrational spectroscopy,^31–33^ with complementation from computational simulation.^20,34,35^ Nevertheless, the investigation is often hampered by the slow kinetics of the transition occurring at low temperatures. Rather than the ideally ordered phase, quite often the orientational glass forms upon cooling, in which the molecular orientations are frozen in a transient state due to insufficient time and slow kinetics.^36^ Even many of the reported “ordered” ice polymorphs contain a substantial degree of disorder.^37,38^ These experimental observations are recognized to indicate the transient states, assuming the existence of the ideally ordered phase.

The existence of partial orders complicates the story. Partially ordered states may be regarded as kinetically arrested disordered forms, in which the molecular configuration has some degree of order. This is observed as a nonhomogeneous distribution of site occupancy of hydrogen atoms (e.g., 0.1 and 0.9) instead of a homogeneous distribution (e.g., 0.5 and 0.5) in diffraction experiments.^27,28,38,39^ In contrast to the kinetic arrest, partial order may also arise as the consequence of thermodynamic equilibration, i.e., in the limit of infinite time. This is the case for ices III and V,^40,41^ potentially also for some of the “ordered” polymorphs.^27,28,37,38,42^ Even though such partially ordered phases carry some arbitrariness in their identification, they experience order–disorder transitions that are discontinuous, first-order transitions as probed from dielectric spectroscopy,^15^ calorimetry,^19,43^ and structural methods.^38^ Nevertheless, their nature remains largely unclear, and further elucidation of the partial disorder remains challenging.

In a microscopic view, the (dis)­ordered states can be described as the distribution of domains of ordered configurations. In the ordered state, only one of these domains persists, where some defects are typically present in real ices (Figure 1A). The disordered state can be described as a homogeneous mixture of many tiny domains of variously ordered configurations (Figure 1C). The sizes of ordered domains in the disordered states are considered to be too small to define the boundaries unambiguously. That is, one ordered configuration may resemble the other very much and might even be a part of it as well. Thus, the ordered state would presuppose finite-sized, homogeneously ordered domains. This finite size may vary by the techniques to probe; e.g., tens of nanometers for X-ray diffraction, nanometers for electron diffraction, and potentially several molecules for computational simulations with theoretical modeling.^34^ In partially ordered states, some specific configurations would be more dominant than the others (Figure 1B). Their distribution may be homogeneous as in the disordered state, or alternatively inhomogeneous, i.e., a mixture of finite-sized domains. Also, the pathway to reach order is unclear: multiple stages of order may appear upon lowering temperature according to computational modeling,^34^ but have actually never been experimentally identified so far.

**Figure 1:**
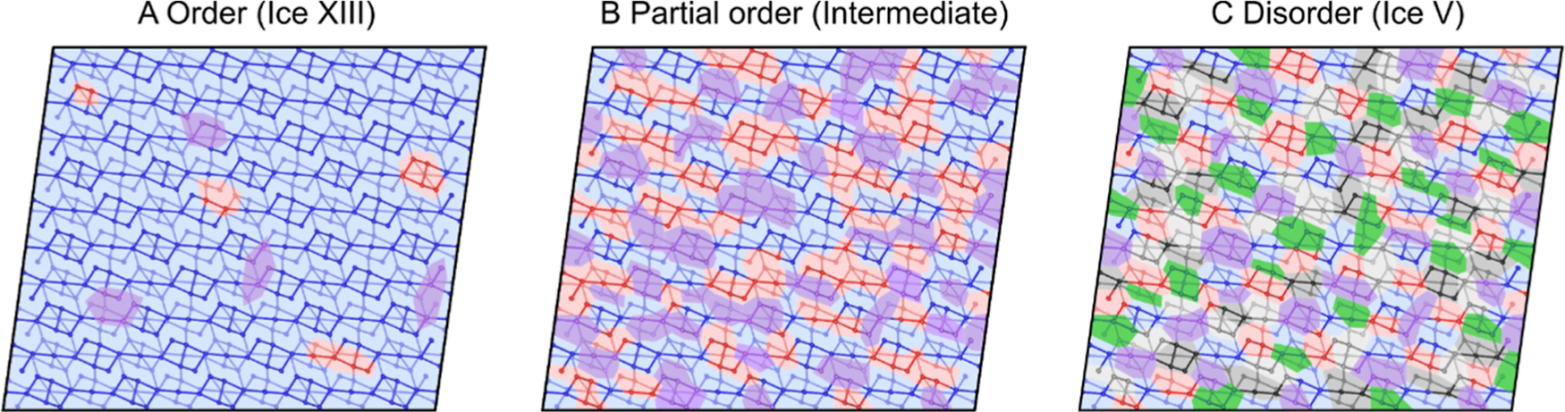
Schematic illustration of (A) ordered, (B) partially ordered, and (C) disordered states, corresponding to ice XIII, the intermediate, and ice V, respectively. Colors represent the types of configuration, i.e., blue for the most stable configuration (ice XIII), red and purple for near-degenerate states, and green, light gray, and dark gray for the others. The ordered state (A) is dominated by blue domains but also contains a small amount of other configurations called defects or residual disorder. The disordered state (C) represents a mixture of tiny domains of ordered configurations, which are hard to distinguish. Thus, the disordered state can be recognized as homogeneous for the time- and/or space-average. A partially-(dis)­ordered state (B) is similarly described as (C), but fewer different configurations appear.

Here, we investigate the calorimetric response of the ice V-XIII pair that we studied in our previous work^44^ as a model case to elucidate the hydrogen ordering process in ice. Ice V is a hydrogen-disordered phase which is thermodynamically stable at around 0.5 GPa and 250 K.^45^ With the aid of dopants such as HCl, ice V can transform into its hydrogen-ordered counterpart, ice XIII.^38^ The maximum enthalpy difference between ices V and XIII is estimated to be ≈250 J/mol (66% of Pauling entropy) at ambient pressure.^19^ The deviation from 100% Pauling entropy is considered to arise from the partial order within disordered ice V^40,41^ and partial disorder within ordered ice XIII^38^ (See Figure 1C). Considering this arbitrariness of partial order, we refer to all the ordered forms as “ordered states” against the equilibrated “disordered” ice V, specifically above 120 K at ambient pressure. On the other hand, “ice XIII” will only refer to the state experimentally observed below 112 K^38^ and the corresponding hypothetical completely ordered form, although residual disorder always remains in the real system even at 12 K.^39^

Ice V shows a significant transformation barrier to ice I at ambient pressure, so that it is kinetically stable and converts to ice I only above 136 K. That is, at ambient pressure, the ice V–XIII transition can be observed reversibly, e.g., when cycling between 90 and 130 K. Previously, we established an isothermal annealing approach to extract simultaneously both thermodynamic and kinetic properties associated with the hydrogen ordering process.^44^ For the natural isotope case, i.e., for H_2_O, we revealed a two-step transition and proposed an intermediate ordered state at 112–120 K that is distinct from both ice V and XIII. This intermediate state provides a rationale for the origin of two endothermic events reported in calorimetry during the disordering of ice XIII and two exothermic events upon ordering of ice V.^19^

In this study, we extend to the deuterated case, i.e., D_2_O, for a comprehensive understanding of the ordering of ice V/XIII. In general, deuteration slows down the kinetics due to deuterium (^2^H, D) being heavier than protium (^1^H). Especially, nuclear quantum effects are suppressed in deuterated samples, most notably quantum tunneling of D atoms to switch places in the D-sublattice.^46^ Thermodynamic properties are also affected by H/D substitution, as both the structure^47–49^ and the phase boundary^50,51^ weakly change. Yet, the thermodynamic isotope effect is rather small compared to the kinetic isotope effect because deuteration does not directly change the chemical properties, i.e., charge and internal energy. Instead, the isotopic substitution affects the vibrational states, which indirectly contribute to the thermodynamics through the phonons.^52^ The slower kinetics of deuterated species is a challenging subject that has so far been neglected in the literature of ordered ices, which instead mostly focuses on the generation of the ordered form and its static structure.^3,27^ The kinetics were often unmonitored. Our isothermal approach overcomes this obstacle and allows access to the ordering behavior more comprehensively, in terms of both thermodynamics and kinetics.^44^ In brief, various ordered states are accessed by isothermal annealing at ambient pressure in the calorimetry apparatus for various anneal temperatures (*T*_anneal_) and anneal times (*t*_anneal_). The time-development of the degree of order and thermal stability against heating is then used to extract kinetic and thermodynamic parameters.

## Methods

Basic procedures followed the previous work.^44^ The protiated solution (0.01 M HCl in H_2_O) was made from concentrated HCl solution (Sigma-Aldrich) diluted with Milli-Q water, and the deuterated solution (0.01 M DCl in D_2_O) was made from concentrated DCl solution (99.5% D, Cambridge Isotope Laboratories) diluted with D_2_O (99.6% D, Eurisotop). Ice V was prepared by crystal–crystal transitions starting from ice I_h_ upon isobaric heating at 0.5 GPa up to ≈250 K using a piston–cylinder cell, following the established procedure.^19,38,44,53^ Afterward, the samples were quenched to 77 K and retrieved at ambient pressure. The hydrogen (dis)­ordering processes of ice V at ambient pressure were investigated by differential scanning calorimetry (DSC).

Before each DSC scan, the sample was once heated to 134 K to erase any kind of hydrogen order from ice XIII and to produce ice V. This ambient-pressure preprocess also eliminates the uncertain factors which potentially occur in the high-pressure preparation or sample storage at liquid nitrogen temperature.^3^ Each series of measurements for a specific annealing temperature (*T*_anneal_: 100–125 K) was performed in a single run without changing the sample. The sample was cooled down at 30 K min^–1^ to *T*_anneal_. After the isothermal annealing for various anneal times (*t*_anneal_), the sample was quenched to 93 K, and the thermal response to hydrogen disordering, an endotherm, was measured upon heating at 30 K min^–1^ to 134 K for H_2_O samples or 138 K for D_2_O samples. Immediately after the measurement up to 138 K, the D_2_O samples were cooled to 134 K to avoid spontaneous decomposition. Such DSC scans were repeated in a single series, changing *t*_anneal_ (=0.1–512 min) with the same *T*_anneal_. The enthalpy changes Δ*H* upon the disordering were evaluated by the integration of the endotherm. Further details are described in Supporting Information Section S1 and ref 44.

## Results

Figure 2 represents the time development of hydrogen order. The degree of order is characterized by the area of the endotherm upon heating (see examples in Figure 2A). This area corresponds to the enthalpy change (Δ*H*) upon the disordering from the ordered states into ice V, which is collected in Figure 2B in dependence on anneal time *t*_anneal_. With the prerequisite of the equilibrium and reversibility, Δ*H* is related to Δ*S*_conf_ by the equation Δ*H* = *T*_tr_Δ*S*_conf_. This typically reaches a plateau value at long times, which we call the maximum enthalpy change Δ*H*_max_. The long-time limit Δ*H*_max_ changes with anneal temperature. At *T*_anneal_ above 120 K, isothermal annealing does not affect Δ*H* (see Figure 2B), and it stays almost constant at ≈140 J mol^–1^ for protiated samples and ≈70 J mol^–1^ for deuterated samples. These values are similar to the directly quenched samples without annealing. The smaller Δ*H*_max_ for deuterated species is attributed to the slower ordering kinetics. This suggests that the ordering took place during quenching in the DSC to 93 K before the heating scan. Hydrogen ordering does not take place, and ice V is the dominant phase above 120 K.

**Figure 2:**
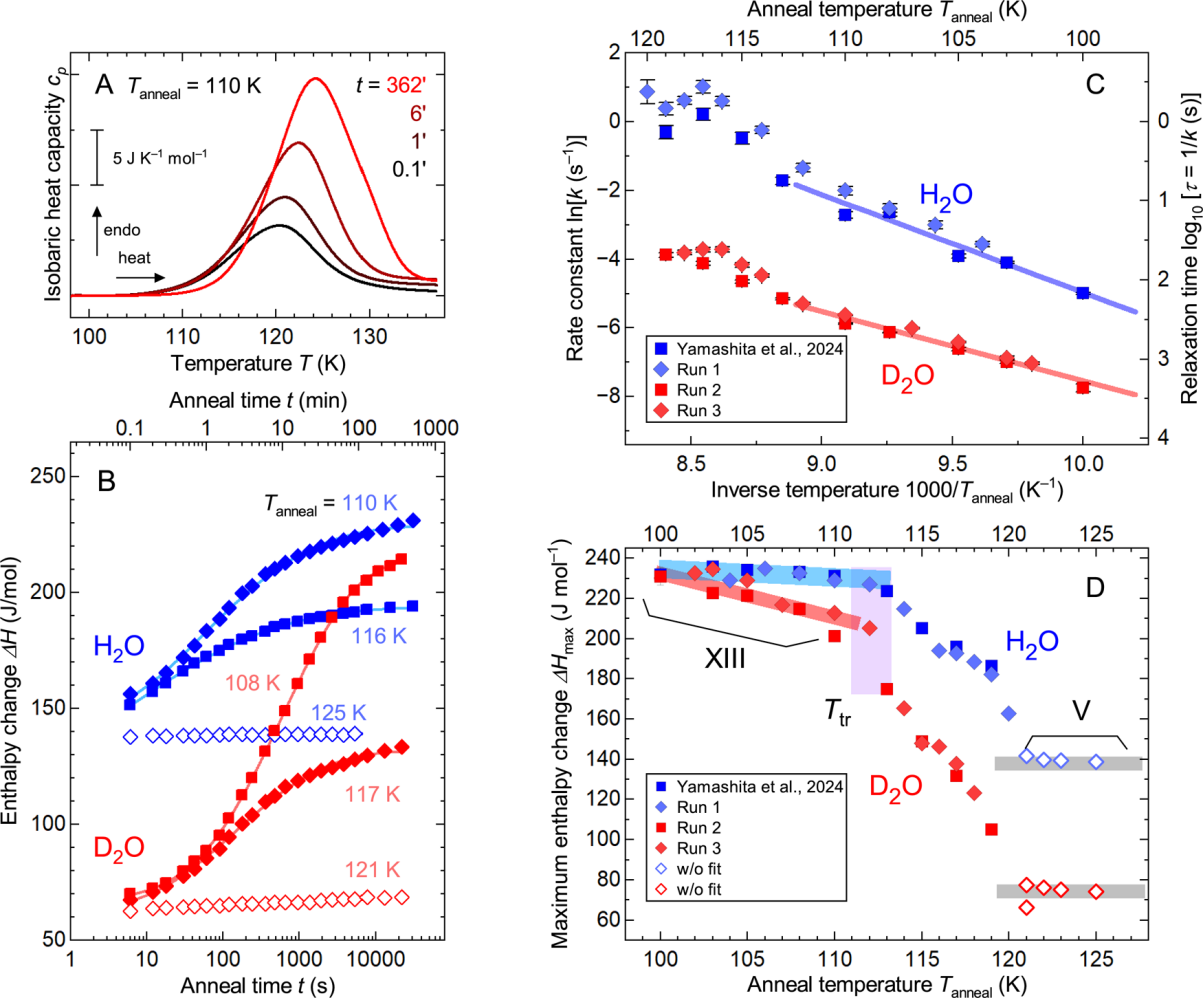
Hydrogen ordering upon isothermal annealing. (A) Representative thermograms for deuterated sample with *T*_anneal_ = 110 K recorded at 30 K min^–1^. All thermograms are baseline-corrected at *T* = 100–103 K. (B) Time evolution of order expressed in terms of enthalpy change Δ*H.* Solid lines are fitted curves using eq 1. (C) Arrhenius plot of rate constant *k*. The relaxation time τ converted from the rate constant by τ = 1/*k* is shown on the right axis. The blue and red lines correspond to the linear regressions of data for *T*_anneal_ = 100–110 K. (D) Maximum enthalpy change Δ*H*_max_ upon disordering into ice V. For *T*_anneal_ > 120 K at which ordering does not proceed, averaged Δ*H* are shown as open diamonds. Gray lines are a guid to the eye. The transition temperature *T*_tr_ is indicated by the shaded region in violet. In (B–D), blue and red symbols correspond to protiated and deuterated samples, respectively. In (C) and (D), both isotopic series contain two sets of samples including the protiated series from the previous work^44^ [Available under CC-BY 4.0; copyright 2024 Yamashita and Loerting].

Below 120 K, Δ*H* is enhanced by isothermal annealing (see Figure 2B), meaning that hydrogen ordering proceeds at such temperatures. Once the ordered states are equilibrated, Δ*H* is expected to be independent of *t*_anneal_ and takes a specific value dependent only on *T*_anneal_. This corresponds to the thermodynamic long-term limit. On the other hand, transient states are encountered at a finite time before the equilibrium, which is observed as the monotonic increase of Δ*H* with *t*_anneal_ until it reaches a plateau. The time evolution at fixed *T*_anneal_ (Figure 2B) is characterized using an exponential-based function

1 $$\Delta H\left(t_{anneal}\right)=\Delta H_{\max}\left(1-\exp\left\{-{\left[k\left(t_{anneal}+\Delta t_{0}\right)\right]}^{n}\right\}\right)$$

This function originates from the modified Johnson–Mehl–Avrami–Kolmogorov (JMAK) equation, widely used for nucleation and growth^54–57^ as well as for kinetic studies on hydrogen (dis)­ordering in ice.^58,59^ This function monotonically increases against *t*_anneal_ with a rate constant (*k*) and asymptotically converges to a specific value (Δ*H*_max_) after a sufficiently long time. The time offset (Δ*t*_0_) compensates for the degree of order that has already developed before reaching *T*_anneal_. The Avrami exponent (*n*) stretches the decay from simple exponential, reflecting the ordering mechanism. Nevertheless, the practical interpretation of experimentally derived *n* values is not straightforward for complicated events (e.g., see ref 60). Thus, this study mainly treats the temperature dependence of the thermodynamic (Δ*H*_max_) and kinetic parameters (*k*).

Figure 2C summarizes the rate constants for the isothermal hydrogen ordering of ice V/XIII made from 0.01 M HCl in H_2_O solution and its deuterated counterpart (D_2_O > 99.5 mol %). Both show similar trends. At lower temperatures, the ordering kinetics get slower as seen in the decrease of *k* for both isotopes (Figure 2C). The deuteration slows down the transition kinetics by a factor of *k*_H_/*k*_D_ ≈ 40 at 110 K. This ratio gets larger at higher temperatures (e.g., *k*_H_/*k*_D_ ≈ 60 at 115 K) and smaller at lower temperatures (e.g., *k*_H_/*k*_D_ ≈ 15 at 100 K). If the hydrogen ordering is governed by a single type of kinetics, the rate constant can be described as thermally activated kinetics in the form of

$$k\left(T\right)=k_{0}\exp\left(-\frac{E_{a}}{k_{B}T}\right)$$ 2

with the pre-exponential factor *k*_0_, the activation energy *E*_a_, and the Boltzmann constant *k*_B_. below *T*_tr_ ∼ 113 K, *k* values follow this Arrhenius behavior, suggesting a straightforward hydrogen ordering from ice V to XIII. In contrast, *k* values are off from the trends near 120 K in both isotopes and are instead less dependent on the temperature. The non-Arrhenius nature at 113–120 K suggests more complex kinetics, e.g., domain-specific kinetics.

The deuterated sample also follows a single type of kinetics below 113 K, i.e., Arrhenius behavior, but shows a slightly shallower slope corresponding to the smaller activation energy. The linear regression for *T*_anneal_ = 100–110 K gives activation energies of 24 (2) kJ mol^–1^ for protiated samples and 16.8 (16) kJ mol^–1^ for the deuterated samples. The differences among data sets are summarized in Table S1. The protiated case is close to the value for local reorientation dynamics in ice V [23 (2) kJ mol^–1^] reported from dielectric spectroscopy,^13^ which implies the global hydrogen ordering in the protiated ice V/XIII is mostly governed by the local reorientation in the disordered matrices. On the other hand, the isotope effect is in opposite trends to the dielectric spectroscopic study,^13^ which observed a larger activation energy for deuterated ice V [28 (2) kJ mol^–1^] than for protiated ice V.

While a direct experimental clue is missing, an orientational glassy scenario may provide a hint. Here, with relaxation time (τ = 1/*k*), “glass transition” temperatures of hydrogen ordering (*T*_g, ordering_) can be defined as the temperature where τ reaches 100 s, in a similar manner to dielectric spectroscopies. This gives *T*_g, ordering_ = 102 K for the protiated samples. This approximately matches the temperatures of orientational glass transition (103 K) from dielectric spectroscopy^13^ and kinetic unfreezing (105 K) upon heating in DSC.^19^ On the other hand, this analysis yields *T*_g, ordering_ = 114 K for the deuterated samples. The ordering into ice XIII occurs below this temperature. It should be noted that our isothermal annealing approach observes a global event whose rate is determined by a combination of processes, including transient back-transformation into ice V as a local structural fluctuation. That is, the ordering phenomenon may be affected by kinetic hindrance, e.g, insufficiency of collaborative reorientation required for the ordering mechanism.

In the pseudoequilibrated condition, Δ*H* reaches a plateau at a specific value dependent only on *T*_anneal_, represented by Δ*H*_max_ (Figure 2D). With decreasing *T*_anneal_ (<120 K), Δ*H*_max_ drastically increases but no longer increases below 113 K, i.e., the highest degree of order has been reached. Little isotope effects are evident at these two temperatures. The kink at 113 K marked in violet in Figure 2D corresponds to *T*_tr_, the temperature which is generally adopted as the phase boundary between ice V and XIII. As discussed in the previous study,^44^ ordered states below *T*_tr_ are assigned to ice XIII. The isotopic comparison following the literature^19^ estimates the maximum Δ*H* between ice V–XIII to be 236 J mol^–1^ (see Supporting Information Section S2), corresponding to 63% of Pauling entropy.^1^

While Δ*H*_max_ is quite similar for both isotopes below *T*_tr_, there is a large difference at intermediate temperatures between *T*_tr_ and 120 K. The deuterated species has significantly lower Δ*H*_max_ with stronger temperature dependence than the protiated species. The origin of this isotope effect can be ascribed to either thermodynamic factors, such as the vibrational energies of partially disordered states, or kinetic factors, such as the mutual conversion of local configurations.

## Discussion

Here, we deduce further details of the proposed intermediate ordered state from another aspect, the endotherm profiles upon heating. Since the DSC measurement probes transient states upon disordering rather than equilibrated states due to the high heating rate (30 K min^–1^ in this study), the endotherm profiles reflect how the disordering takes place and how thermally stable ordered states are after isothermal annealing. For example, protiated samples show narrower endotherms than deuterated samples, attributed to the slow dynamics of D_2_O^19^ [Full-Width at Half Maximum: fwhm = 7.6 ± 0.1 K (protiated) and 10.3 ± 0.1 K (deuterated) for samples continuously cooled at 30 K min^–1^; see Supporting Information Figure S4 inset].

As a general idea, well-ordered structures transiently survive up to higher temperatures because of the locked molecular reorientations in the ordered configuration.^13^ The ordered configuration is at a potential minimum on the energy landscape, and it needs to overcome a high energy barrier due to the interlocking collective reorientation of the molecules compared to less-ordered (disordered) states. The endotherm position shows the trend changes of time evolution at *T*_anneal_ = 120 K and *T*_tr_, as discussed in our previous work.^43^ In brief, the peak position does not change for *T*_anneal_ > 120 K, shifts to higher temperatures by annealing, but reaches a plateau in ≈10 min for *T*_anneal_ between 120 K and *T*_tr_, and continues to change over an hour for *T*_anneal_ below *T*_tr_. See Supporting Information Section S6 for the details.

Figure 3 shows the relation between the endotherm shift Δ*T*_top_ and the enthalpy change Δ*H*. Here, Δ*T*_top_ is the difference of the endotherm positions from the reference case without annealing (See Supporting Information S3 for the details). Regardless of isotopes and the anneal temperature, Δ*H* and Δ*T*_top_ show linear relations, except for Δ*H* above 190 J mol^–1^. The intermediate ordered state (between *T*_tr_ and 120 K) stays on this trend while reaching the equilibrium mostly after 10 min or less than an hour for both isotopes. The ordered states below *T*_tr_ also follow the linear trend in the initial part of annealing where Δ*H* is below 190 J mol^–1^. This common feature implies that a similar ordering process takes place for all the temperature conditions for the initial part of ordering. As a simple scenario, the introduction and development of ordered domains (See Figure 1A) leads to the thermal stability of the ordered states. In other words, the ordered states can be explained simply as a linear combination of hypothetical binary states of disordered and ordered forms. This resembles the colligative behavior of the melting temperature depression of dilute solutions.

**Figure 3:**
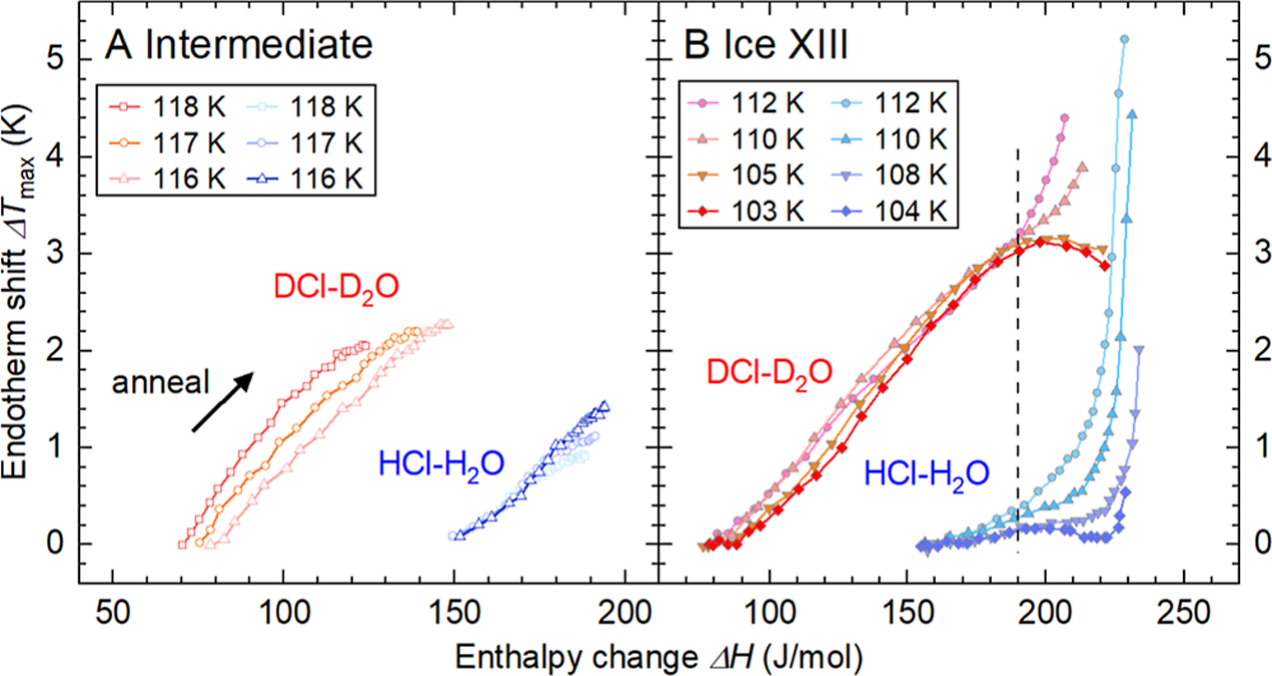
Endotherm shifts Δ*T*_max_ against enthalpy change Δ*H* for (A) intermediate ordered states (*T*_anneal_ = 116–118 K) and (B) ice XIII (*T*_anneal_ < 113 K). Blue and red symbols correspond to protiated and deuterated samples, respectively. The dashed line in B shows the threshold boundary where the Δ*T*_max_–Δ*H* relations divert from the linear trend.

On the other hand, Δ*T*_top_ diverges from the linear trend for Δ*H* above 190 J mol^–1^, which implies the system is more governed by the ordered structures, and the hypothetical ordered form extrapolated from the less-ordered states does not appropriately reflect the disordering behavior of the well-ordered phase. The threshold for the remnant disorder can be estimated as the difference between this threshold and the maximum enthalpy difference (236 J mol^–1^), giving a value of 46 J mol^–1^, corresponding to 12% of Pauling entropy with *T*_tr_ ∼ 113 K. Leaving aside the difference between the network topology, this threshold of 88% degree of order can be marked as a guide to investigate the hydrogen-ordered phases. If the degree of order is not as high as this threshold, the observed physicochemical properties of hydrogen-ordered states may not reflect ideal “ordered phases” as for a single configuration, but be affected by the coexisting other configurations, recognized as disorder. This also explains the overestimate of the maximum enthalpy difference (250 J mol^–1^) in the literature.^19^

The details of the differences in the diverging trends of Δ*T*_top_ for Δ*H* > 190 J mol^–1^ are not clear. With the assumption that the well-ordered structure is more thermally stable as found for *T*_anneal_ = 110 K of protiated samples, the Δ*T*_top_ decrease would correspond to the introduction of defects in order to accomplish the global ordering of the system (See Figure 1A). For example, Δ*T*_top_ once decreases at Δ*H* = 190–220 J mol^–1^ for *T*_anneal_ = 104 K of protiated samples but also shows a sign of upturn to drastic increase above Δ*H* = 220 J mol^–1^ (Figure 3B), which may reach as high as ≈5 K if sufficiently long anneal time is provided to remove the remnant disorder. For *T*_anneal_ = 103 K of deuterated samples, a decreasing trend is found above Δ*H* = 190 J mol^–1^, but no upturn is observed even after the ≈8 h of annealing. Even the longest annealing in this study is not long enough for the slow kinetics. In a similar manner to the protiated series, Δ*T*_top_ may turn to increase as well if much longer anneal time is provided, like a geological time scale in icy bodies.

Considering the trend change in Δ*T*_top_, the ordering process can be separated into two stages (Figure 4): the formation of locally ordered structures from a disordered matrix, followed by conversion into the ordered states toward an enthalpically favored configuration (i.e., ice XIII). The former stage involves the formation of near-degenerate but nonideally ordered configurations different from ice XIII, corresponding to the conversion from the states described in Figure 1C to those in Figure 1B. These can be regarded as residual disorder against the optimal configuration. The latter stage removes such residual disorder, reaching almost complete order (See Figure 1A), but takes more time than the first stage. This second stage would correspond to the observation in our previous study of ice XII-XIV, where slow ordering proceeds during cryo-storage at 77 K, so that even after years, the order continues to enhance.^3^

**Figure 4:**
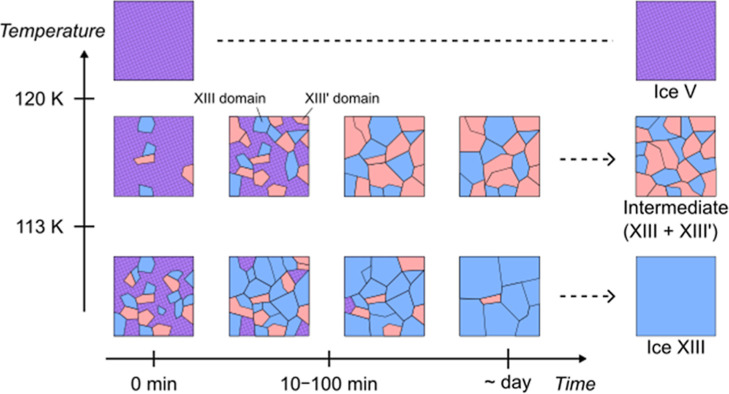
Schematic illustration of a hypothetical model as the development of ordered domains with time at three different temperature regimes. Blue and red colors correspond to the domains of ordered ice XIII and ordered ice XIII’. Here, ice XIII’ indicates near-degenerate configurations from the possible ordered structures different from ice XIII. Violet regions correspond to the disordered part recognized as ice V (See Figure 1 and the main text). Above 120 K, ice V remains disordered. Below 120 K, ordering proceeds similarly up to 10–100 min. At intermediate temperatures, the system is equilibrated as mixtures of ice XIII and XIII’ after 10 min, retaining mutual conversion among configurations. Below *T*_tr_ (∼113 K), ordering proceeds further, gradually reaching the ideally ordered structure (ice XIII).

The second stage was little featured for the intermediate ordered states. One scenario is that the further ordering into a single configuration (i.e., ice XIII) does not take place as the dominant process. Instead, mutual conversions among different ordered configurations take place in the second stage, owing to the entropic compensation between near-degenerate states. This means the intermediate ordered state is a result of a dynamical configurational mixture (see Figure 1B). In the dynamically converting system, the equilibrium distribution of population is determined by the ratio of the conversion rates among the configurations. For example, the equilibrium ratio between domains of types A and B is then determined as A/B = *k*_2_/*k*_1_ from the rate constants *k*_1_ and *k*_2_ for the reverse conversion. If the isotope substitution affects the conversion rates differently, this will give the isotope effects on the resultant domain ratio. Given that isotopic substitution largely affects the kinetics rather than the thermodynamics, this domain equilibrium explains the large isotope effect on Δ*H*_max_ of the intermediate as well (Figure 2D).

## Conclusions

These findings highlight the complexity of the hydrogen (dis)­ordering phenomena and the difficulty of their elucidation from simple point-by-point measurements. As seen from the tremendous differences in terms of ordering at two different temperatures (e.g., 113 K vs. 120 K), the actual ordering process is far from a simple picture in which one disordered form continuously develops into an ordered form, unless it gets stuck in the orientational glass. Our study here represents a case of dynamic equilibrium of ordered domains. Yet, this observation might also apply to other ice polymorphs. The recent observation of hydrogen sublattice polymorphism from ice VI^27,28^ may represent one manifestation of the domain conversion complexity in the order–disorder transitions.

## Supplementary Material



## Notes

Complementary data and description including (S1) details of experimental procedures, (S2) Temperature dependence of Avrami exponent, (S3) Degree of order independent of isothermal annealing, (S4) Estimation of maximum enthalpy difference between ice V and XIII, (S5) Comparison of activation energy among data sets, and (S6) time dependence of endotherm shifts (PDF)
