# Supplementary material for: Isotope Effect on the Hydrogen Ordering from Ice V to Ice XIII via a Partially Ordered Intermediate
Source: J Phys Chem B. 2026 Jun 13;130(28):7242–9. doi: 10.1021/acs.jpcb.6c01462 (PMC13383724; doi:10.1021/acs.jpcb.6c01462)
Supplement: Supplementary file 1 [file jp6c01462_si_001.pdf]

Supporting Information

Isotope Effect on the Hydrogen Ordering from Ice V to Ice XIII via a Partially Ordered Intermediate

Keishiro Yamashita[†,‡,*], Thomas Loerting[†]

[†]Institute of Physical Chemistry, University of Innsbruck, Innrain 52c, 6020 Innsbruck, Austria

[‡]SUPA, School of Physics and Astronomy and Centre for Science at Extreme Conditions, The University of Edinburgh, Edinburgh EH9 3JZ, U.K.

*Email: kyamashi@ed.ac.uk

S1 Details of experimental procedures

Sample preparation

0.01 M HCl or DCl solution was poured into an indium container precooled at liquid nitrogen temperature. The sample was pressurized under liquid nitrogen temperature, using a high-pressure piston cylinder with an 8 mm and 10 mm bore for deuterated and protiated samples, respectively, together with a commercial "universal material testing machine" (Zwick, model BZ100/TL3S).[1–3] Ice V finally forms as the thermodynamically stable phase *via* the solid-solid transitions (I → III/IX → II → V[4,5]) upon isobaric heating at 0.5 GPa up to *ca.* 250 K. Afterwards, the samples were quenched to 77 K and retrieved at ambient pressure.

Calorimetric analysis

The enthalpy changes ΔH, determined as the area of endotherm upon disordering, were analyzed by Equation 1 as described in the main text,

$$\Delta H(t_{\mathrm{anneal}}) = \Delta H_{\mathrm{max}}\,(1 - \exp\{-[k(t_{\mathrm{anneal}} + \Delta t_0)]^n\}). \quad (1)$$

Here, the variables (ΔH_{max}, k, Δt_0, and n) are fitted as parameters for measured ΔH against t_{anneal}. There are several factors affecting the observed parameters and errors. Based on the large number of experiments, we settled the error of the fitting parameters in eq 1. The error bars are mostly smaller than the symbol size used in Figure 2. The realistic error derives mainly from the scatter between different sample preparations and calorimetry experiments, which are relevant for determining the activation energies from the Arrhenius relation (Equation 2).

S2 Temperature dependence of Avrami exponent

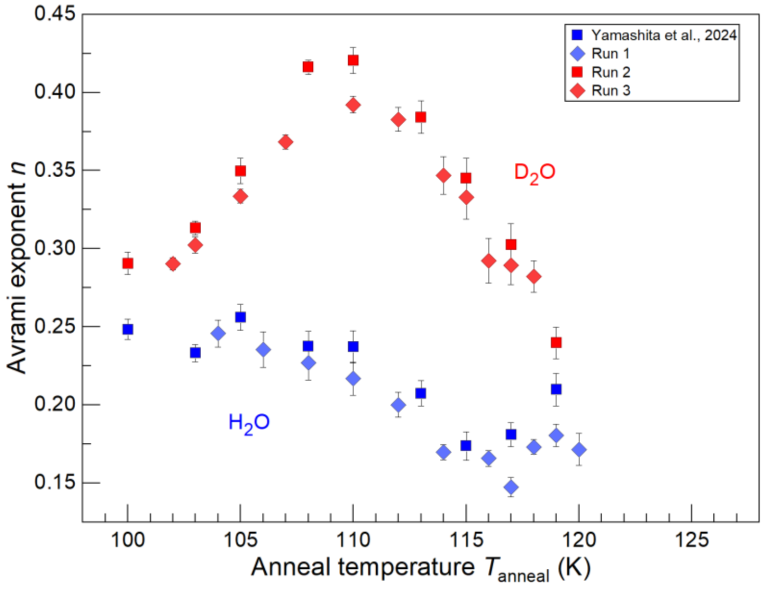

Figure S1 Avrami exponent *n* in accordance with eq 1 described in the main text.

S3 Degree of order independent of isothermal annealing

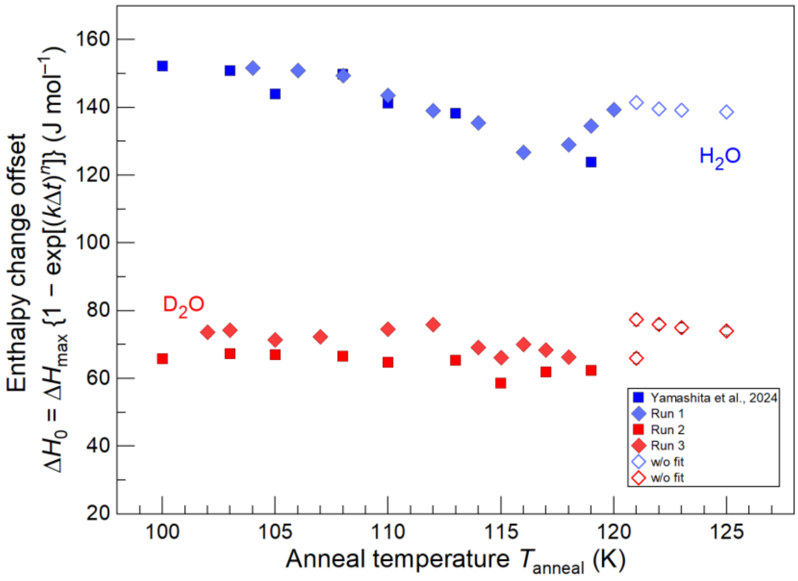

Figure S2 Enthalpy offset $\Delta H_0 = \Delta H_{max}$ $\{1 - \exp[-(k\Delta t_0)^n]\}$ in accordance with Eq. 1 described in the main text. This value would correspond to the degree of order independent of the isothermal annealing, which can originate from the ordering before the isothermal annealing. For $T_{anneal} > 120$ K, averaged ΔH as measured are also described in open diamonds for comparison with the case in which isothermal annealing does not enhance the ordering.

S4 Estimation of maximum enthalpy difference between ice V and XIII

Following the literature,[6] isotopic comparisons give a hint of the upper threshold of enthalpy difference upon hydrogen ordering. Figure S3 shows the average of ΔH_{max} against the isotopic difference. In the same manner as the description in the main text, ice XIII (T_{anneal} < 113 K) and the intermediate (T_{anneal} > 113 K) show the gap of isotopic difference. In a completely ordered structure, the isotopic difference is expected to be negligible. The upper ΔH threshold can be estimated as the intercept on the *y*-axis. The extrapolation for ice XIII gives 236 J mol^{-1}, slightly smaller than the estimate in the literature (250 J mol^{-1}). The reported study[6] used a continuous cooling approach for the ordering, and may be affected by the intermediate ordered state. Also, the isotopic difference was larger than 30 J mol^{-1}. These may have led to an overestimation.

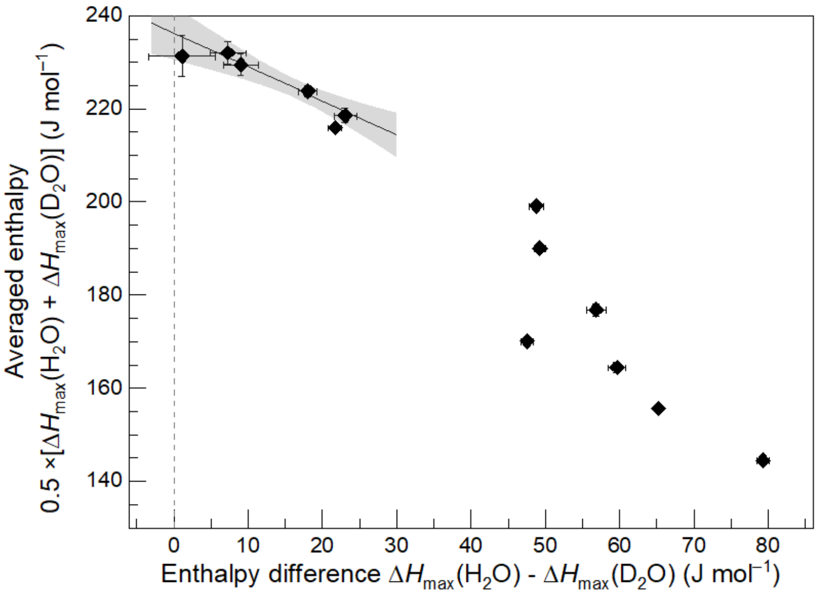

Figure S3 Visualization of the isotope effect in a plot of the average of the maximum enthalpy difference *vs*. difference from the data in Figure 2a and literature.[7] Errors are from fitting using eq 1. The solid line and gray shade correspond to the linear regression for ice XII data (T_{anneal} < 113 K) and 95% confidence band.

S5 Comparison of activation energy among data sets

Table S1 Activation energies of hydrogen ordering of ice V/XIII with 0.01 M HCl or DCl. The linear regressions of the rate constant (*k*) with Eq. 2 used data at T_{anneal} = 100–110 K of individual batches.

	Data sets	Activation energy E_a (kJ mol^{-1})
HCl-H_2O	Ref.[7] + Run 1	24 (2)
	Ref.[7]	22 (2)
	Run 1	24.5 (3)
DCl-D_2O	Run 2 + Run 3	16.8 (16)
	Run 2	17.0 (10)
	Run 3	16.9 (9)

S6 Time dependence of endotherm shifts

Figure S4 shows the representative changes of endotherm shift (ΔT_{max}) through the isotherm annealing (Other data are shown in Figure S4). Here, ΔT_{max} is defined as the difference of the peak tops compared to the reference case (peak top at T_{max} = 119–120 K) of continuous cooling at 30 K min^{-1} without annealing in the same DSC run (same definition as ref.[7]). At T_{anneal} > 120 K, annealing does not affect ΔT_{max} and ΔT_{max} stays comparable to or slightly lower than the continuous cooling at 30 K min^{-1} in the same manner as ΔH_{max} (Figure 2). Instead, ΔT_{max} generally increases according to the annealing below 120 K for both isotopes (Figure S4), except for the protiated samples with T_{anneal} = 100 K, attributed to the glassy bahavior in the previous work.[7] Their time-dependent developments differ by t_{anneal} and T_{anneal}. Their understanding needs to be discussed, separating the anneal time into the beginning and later parts, as discussed in the main text.

In the beginning parts (t_{anneal} < 10 min), ΔT_{max} monotonically increases according to the isothermal annealing. In this time region, ΔT_{max} is in almost linear relation to ΔH (Figure S4). This implies that this ordering part is attributed more to the straight ordering developments from ice V. Between the isotopes, deuterated samples exhibit a larger ΔT_{max} than protiated cases. This may be related to the slower dynamics in deuterated species, as seen in the broad endotherms.

The longer t_{anneal} shows distinction among T_{anneal}. In higher-temperature cases (T_{anneal} = 115–120 K), ΔT_{max} reaches a plateau of 1–3 K mostly after 10 min for both isotopes. This is attributed to the equilibration of the

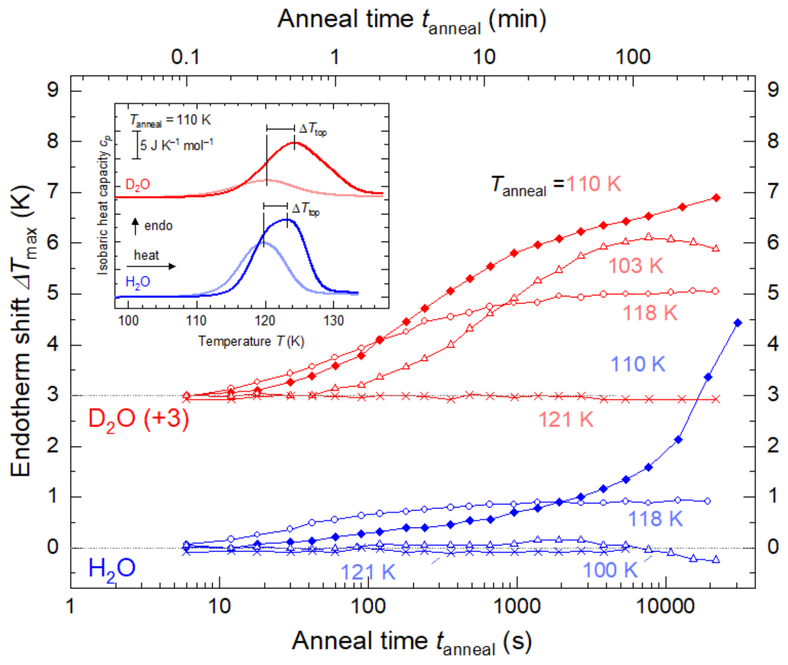

Figure S4 Time dependence of endotherm shift ΔT_{top} of ice V/XIII against isothermal annealing at representative anneal temperature T_{anneal}. The disordering endotherms were recorded upon heating at 30 K min^{-1}. Blue and red plots correspond to protiated and deuterated samples, respectively. Plots for deuterated samples are shown with offset of 3 K. Inset shows the graphical definition of ΔT_{top} with an example of T_{anneal} = 110 K (blue for protiated sample with anneal time t_{anneal} = 322 min and red for deuterated samples with t_{anneal} = 362 min), as peak top difference from continuously cooled case at 30 K min^{-1} (pale colors). Representative thermograms are aligned by subtracting a linear baseline before the endotherms for clarity.

ordered states as seen in the decreasing trends of plateau ΔT_{max}, lower T_{anneal}, consistent with the trend of ΔH_{max} (Figure 2). The behaviour below T_{anneal} = 115 K gets more complicated in the later parts.

In many cases, such as T_{anneal} = 110 K for both isotopes (Figure S4), the ΔT_{max} increases monotonically. This can be attributed to the formation of well-ordered structures or further removal of configurational defects from the ordered matrix of ice XIII.[7] In protiated samples, ΔT_{max} increases drastically after $t_{anneal} \approx 10$ min and can reach even +5 K (also see Figure 4 in ref.[7]). This drastic increase is characterised by the occurrence of a higher-temperature feature of endotherm.[7] Instead, the higher-temperature feature was not obvious, and the increase of ΔH_{top} gets more moderate in the deuterated cases. Nevertheless, ΔT_{max} continues to increase and reaches +4 K. The less time-dependent ΔT_{max} increase of deuterated species would be related to the restriction of collective reorientations in locally ordered configurations, as discussed for activation energy.

As represented by T_{anneal} = 100 K (protiated) or 103 K (deuterated) in Figure S4, some of ΔT_{max} decrease by 0.5–1 K from the largest ΔT_{max} after t_{anneal} = 100 min. In the previous work on the protiated samples,[7] this decreasing trend after isothermal annealing was assigned to the glassy behavior based on the negative ΔT_{max} and correspondence to the orientational glass transition at 102–103 K.[6,8] That is, remaining defects due to the kinetic freezing would promote the disordering. This partly fits with the case of deuterated species, considering its pseudo-glassy feature below 114 K deduced from k, which also indicates a higher threshold temperature than protiated cases.

References for Supplemental Information

(1) Seidl, M.; Amann-Winkel, K.; Handle, P. H.; Zifferer, G.; Loerting, T. From Parallel to Single Crystallization Kinetics in High-Density Amorphous Ice. *Phys. Rev. B - Condens. Matter Mater. Phys.* **2013**, *88* (17), 1–6. https://doi.org/10.1103/PhysRevB.88.174105.

(2) Seidl, M.; Fayter, A.; Stern, J. N.; Zifferer, G.; Loerting, T. Shrinking Water's No Man's Land by Lifting Its Low-Temperature Boundary. *Phys. Rev. B - Condens. Matter Mater. Phys.* **2015**, *91* (14), 1–14. https://doi.org/10.1103/PhysRevB.91.144201.

(3) Seidl, M.; Fayter, A.; Stern, J. N.; Amann-winkel, K.; Bauer, M.; Loerting, T. High-Performance Dilatometry under Extreme Conditions. *Proc. 6th Zwick Acad. Day 2015* **2015**.

(4) Whalley, E. Energies of the Phases of Ice at Zero Temperature and Pressure. *J. Chem. Phys.* **1984**, *81* (9), 4087–4092. https://doi.org/10.1063/1.448153.

(5) Petrenko, V. F.; Whitworth, R. W. *Physics of Ice*; Oxford University Press: Oxford, 2002. https://doi.org/10.1093/acprof:oso/9780198518945.001.0001.

(6) Salzmann, C. G.; Radaelli, P. G.; Finney, J. L.; Mayer, E. A Calorimetric Study on the Low Temperature Dynamics of Doped Ice V and Its Reversible Phase Transition to Hydrogen Ordered Ice XIII. *Phys. Chem. Chem. Phys.* **2008**, *10* (41), 6313–6324. https://doi.org/10.1039/b808386j.

(7) Yamashita, K.; Loerting, T. Thermodynamically Stable Intermediate in the Course of Hydrogen Ordering from Ice V to Ice XIII. *J. Phys. Chem. Lett.* **2024**, *15* (4), 1181–1187. https://doi.org/10.1021/acs.jpclett.3c03411.

(8) Köster, K. W.; Raidt, A.; Fuentes-Landete, V.; Gainaru, C.; Loerting, T.; Böhmer, R. Doping-Enhanced Dipolar Dynamics in Ice v as a Precursor of Hydrogen Ordering in Ice XIII. *Phys. Rev. B* **2016**, *94* (18), 184306. https://doi.org/10.1103/PhysRevB.94.184306.
